# Structural and functional changes to lymph nodes in ageing mice

**DOI:** 10.1111/imm.12727

**Published:** 2017-03-16

**Authors:** Vivian M. Turner, Neil A. Mabbott

**Affiliations:** ^1^ The Roslin Institute and Royal (Dick) School of Veterinary SciencesUniversity of EdinburghMidlothianUK

**Keywords:** ageing, follicular dendritic cells, lymph node, macrophages, stromal cells

## Abstract

Lymph nodes (LN) are secondary lymphoid organs spread throughout the lymphatic system. They function to filter pathogenic material from the lymphatic fluid to maintain the health of the organism. Subcapsular sinus macrophages (SCSM) are among the first‐responders within the LN due to their strategic location within the subcapsular sinus region. These macrophages aid the delivery of immune complexes to B cells and follicular dendritic cells (FDC) within the LN. Here we show an increase in SCSM and other macrophage populations within aged LN. However, immune complex uptake by macrophages within LN was not altered with age, nor was immune complex uptake by B cells. LN stromal cell populations, important in immune responses and the localization and survival of leucocytes, were altered in their representation and distribution in aged LN. In particular, FDC regions were decreased in size and had decreased chemokine CXCL13 expression. Furthermore, the retention of immune complexes by FDC was decreased in aged LN at 24 hr post‐injection. As FDC are important in the maintenance of germinal centre responses, the decreased retention of immune complex in aged LN may contribute to the reduced germinal centre responses observed in aged mice.

**Abbreviations**
BEC: blood endothelial cell
DN: double‐negative
FDC: follicular dendritic cell
FRC: fibroblastic reticular cell
GC: germinal centre
IC: immune complex
IHC: immunohistochemistry
LEC: lymphatic endothelial cells
LN: lymph node
MCM: medullary cord macrophage
MSM: medullary sinus macrophage
PE: phycoerytherin
SCSM: subcapsular sinus macrophage

## Introduction

Lymph nodes (LN) are distributed throughout the body, connected by the lymphatic system. Lymphatic fluid, carrying pathogens and antigens, enters the LN into a cavity under the LN capsule. This subcapsular sinus region is home to a specialized subset of cells termed subcapsular sinus macrophages (SCSM). These macrophages capture pathogens and antigens in the form of antigen‐containing immune complexes (IC) from the lymph, resulting in their degradation or surface retention. The subsequent relay of these antigens to B cells in the neighbouring follicles activates the B cells to produce antibodies.^1^, ^2^ The activated B cells migrate towards, and pass on these antigens to, the follicular dendritic cells (FDC) within the B‐cell follicles.^3^ The FDC rapidly internalize these antigenic IC, then undergo cyclic rounds of IC expression on their surface, encouraging the formation of germinal centres (GC) and adaptive immune responses.^3^ Stromal cells play important roles in this process by coordinating the movement of B and T cells along FDC and fibroblastic reticular cells (FRC), respectively, when migrating around the LN.^4^

In humans and mice, ageing LN have decreases in T‐cell populations, display dysregulated interactions between T and B cells, altered lymphocyte movement, decreased number and size of GC and altered structural organization.^5^, ^6^, ^7^, ^8^, ^9^, ^10^, ^11^, ^12^ Aged FDC have also been shown to have decreased antigen retention in the days after the initiation of an immune response.^7^, ^8^, ^13^ However, little is known of the effects of ageing on macrophage and stromal cell populations in the aged LN.^14^ We therefore set out to characterize the changes that occur in these populations in aged LN, and also to determine whether ageing impacted on IC uptake and relay in the LN during the first 24 hr after immunization.

## Material and methods

### Mice

Female C57BL/6J mice were purchased from Charles River UK and Charles River France. Mice were maintained in‐house under specific pathogen‐free conditions. All experimental procedures were approved by The Roslin Institute's Ethical Review Committee. Experiments were conducted under the authority of the UK Home Office Animals (Scientific Procedures) Act 1986. Young mice were used at 7–12 weeks old, whereas aged mice were used at 18–21 months old.

### Flow cytometry

For analysis of T and B cells, cervical, axillary, brachial and inguinal LN were pooled and made into a single‐cell suspension by passing through a 0·7‐μm cell strainer (Thermo Fisher Scientific, Waltham, MA) and processed on ice during staining. An LSR Fortessa with diva software (BD Biosciences, Oxford, UK) was used for flow cytometry. Data were analysed using flowjo (FlowJo, LLC, Ashland, OR). Cells are gated on lymphocytes then doublets before quantification.

### Extraction of stromal and macrophage LN populations for flow cytometry

Cervical, axillary, brachial and inguinal LN were removed from euthanized mice and broken up with 25G needles. LN were resuspended in 750 μl of 1 mg/ml Collagenase IV (Sigma‐Aldrich, St Louis, MO) with 40 μg/ml DNAse I (Roche, Basel, Switzerland) and placed into a 37° water bath. Vigorous pipetting was used to break up nodes at regular intervals. Once digested (approximately 30 min) suspension was moved into PBS with 5 mm EDTA and 0·1% bovine serum albumin and kept in this solution during FACS staining and data collection. Greater than 95% viability, as determined by trypan blue exclusion, was achieved using this method. For stromal cell analysis, cells were fixed in formalin after extracellular staining then permeabilized overnight with PBS containing 0·2% Tween‐20 and 0·02% sodium azide before intracellular staining for propidium iodide (PI) (eBiosciences, San Diego, CA). Stromal cells were identified using the gating depicted in Figure 3(a). Forward scatter and side scatter were used to remove dead cells, doublets and debris. CD45 and TER119 were used to remove lymphocytes and red blood cells, respectively. Propidium iodide confirmed DNA content of the cells and exclusion of further debris.

### Immunofluorescence

Inguinal LN were used for all immunofluorescent images and are representative of six mice per group. Frozen sections 6–8 μm thick were fixed in ice‐cold acetone, rehydrated in PBS and blocked with normal horse serum before antibody application. Dako (Agilent, Santa Clara, CA) fluorescent mounting medium was used to apply coverslips before image acquisition. A Zeiss LSM5 Pascal upright microscope or Zeiss LSM 710 inverted confocal microscope (Carl Zeiss, Oberkochen, Germany) with zen software (Rochdale, UK) was used for image collection. Images were analysed using image J software (NIH). To determine average FDC size and SCSM depth between 4 and 11 images were analysed per LN, depending on the size of the LN.

### Antibodies

The following antibodies were purchased from BioLegend (San Diego, CA): anti‐CD3e (145‐2C11), anti‐CD4 (RM4‐5), anti‐CD11b (M1/70), anti‐CD31 (390), anti‐CD45 (30‐F11), anti‐CD45R/B220 (RA3‐6B2), anti‐CD169 (3D6.112), anti‐F4/80 (BM8), anti‐MAdCAM‐1 (MECA‐367), anti‐Syrian hamster biotin (Poly4056) and anti‐TER119 (TER119). Anti‐CD35 (8C12) and anti‐CD16/32 (2.4G2) were purchased from BD Biosciences. Streptavidin Alexa Fluor 594, Alexa Fluor 488 and Alexa Fluor 647 were purchased from ThermoFisher Scientific. Anti‐podoplanin/GP38 (8.1.1) was purchased from the Developmental Studies Hybridoma Bank (Iowa City, IA).

### *In vivo* immune complex tracking

An established protocol was adopted to compare movement of phycoerythrin (PE)–IC complexes within young and aged inguinal LN.^1^ Briefly, mice were given 2 mg of polyclonal rabbit IgG anti‐PE (GTX27011; GeneTEX, Irvine, CA) intraperitoneally. After 16 hr mice were sedated with isoflurane and given 5 μg of PE (P801; Invitrogen, Carlsbad, CA) subcutaneously into each side of their flank. At 2, 8 and 24 hr after subcutaneous injections mice were killed and one inguinal LN was taken for flow cytometric analysis while the other was snap frozen in OCT (Biotek, Winooski, VT).

### Imaris analysis of IC trapping

Ten‐micrometre thick sections were imaged on a Zeiss LSM710 confocal microscope. Scanning was sequential with a 1·58‐microsecond dwell time. Images were captured at a resolution of 1024 × 1024 pixels using a 63× (NA 1·4) objective, a 1 × software zoom and a *z*‐step size of 0·44 μm. Images were saved in the lsm format and image analysis was performed using imaris 8.2.1 software (Bitplane, Belfast, UK) and matlab (Natick, MA) Image analysis was adapted from a published protocol.^15^ Two FDC networks were imaged per mouse and graphs show individual images as points.

### Statistical analysis

graphpad prism (GraphPad software, La Jolla, CA) was used for all statistical analyses. Median values are shown on graphs as horizontal lines, and the Mann–Whitney *U*‐test was used to determine significance.

## Results

### Aged mice have a decreased representation and altered organization of lymphocytes in LN

**Figure 1:**
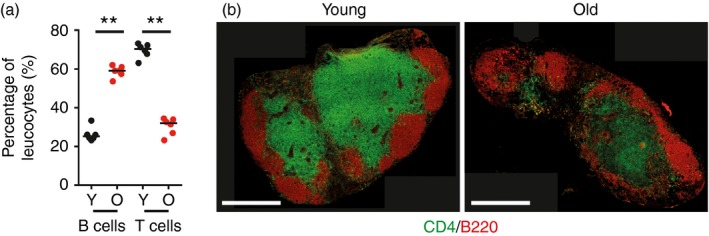
Aged mice have a decreased T‐cell population in their lymph nodes (LN). T and B lymphocyte populations were analysed by flow cytometry and immunostaining in young and aged mice. (a) Flow cytometric analysis of T‐cell (CD3e^+^) and B‐cell (B220^+^) populations from young and aged LN, pooled from two experiments with three mice per group in each experiment. (b) Histological staining of T‐cell (CD4, green) and B‐cell (B220, red) populations in the inguinal LN of young and aged mice, representative of six mice per group. **P < 0·01. Scale bars represent 500 μm.

Alterations to T‐cell and B‐cell populations in LN from ageing humans have been demonstrated,^6^ so it was first pertinent to confirm whether similar changes were observed in the LN of aged mice. Flow cytometric analysis demonstrated a relative decrease in T cells and relative increase in B‐cell populations (Fig. 1a). Immunohistochemical (IHC) analysis confirmed that whereas the B‐cell population was maintained in the LN of aged mice, there was a loss of T cells (Fig. 1b). However, a disruption to the structural localization of B cells was evident, with less defined follicular regions in LN from aged mice compared with young mice (Fig. 1b).

### Aged mice have increased LN macrophage populations

**Figure 2:**
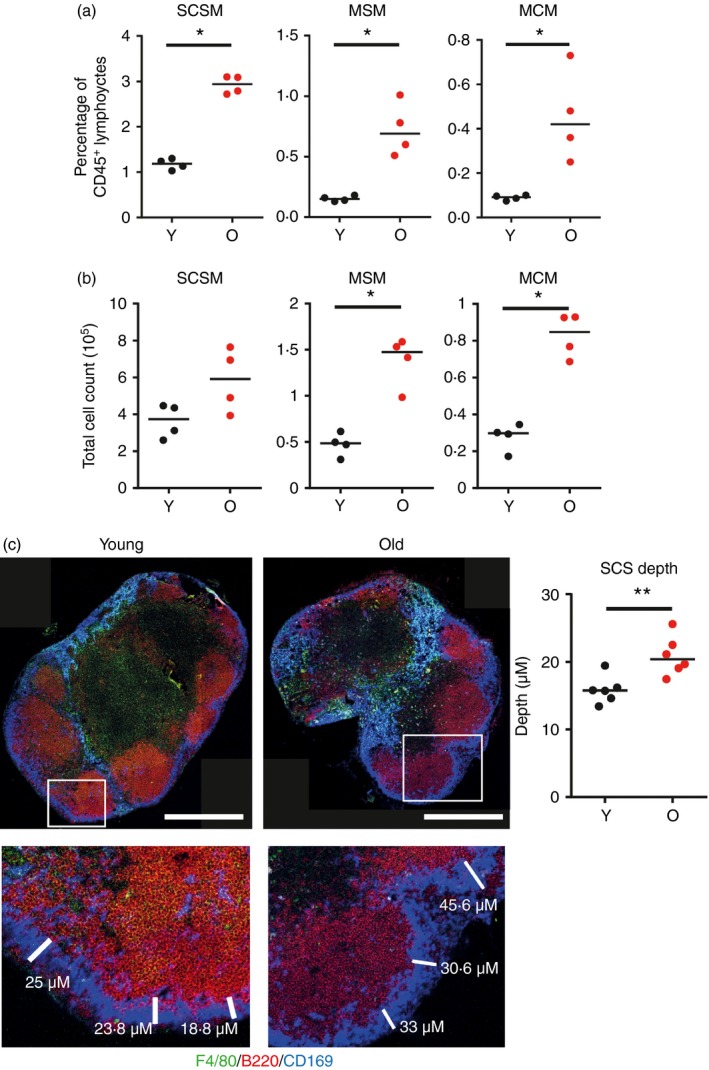
Aged lymph nodes (LN) have an increased representation of macrophages. Macrophage populations were analysed via flow cytometry and immunostaining in young and aged LN. Flow cytometric analysis of subcapsular sinus macrophages (SCSM; CD45^+^, CD11b^+^, CD169^+^, F4/80^−^), medullary sinus macrophages (MSM; CD45^+^, CD11b^+^, CD169^+^, F4/80^+^) and medullary cord macrophages (MCM; CD45^+^, CD11b^+^, CD169^−^,F4/80^+^) was performed. The percentage representation (a) and total cell count (b) of the populations are shown and are representative of three experiments with three to four mice per group in each repeat. (c) Histological analysis of macrophage populations in the inguinal LN from young and aged mice, representative of six mice per group. SCSM are CD169^+^, F4/80^−^ (blue), MSM are CD169^+^, F4/80^+^ (cyan) and MCM are CD169^−^,F4/80^+^ (green). Measurement of the depth of SCS macrophages from the capsule demonstrates an increased depth in aged LN. **P < 0·01; *P < 0·05. Scale bars represent 500 μm.

Next macrophage populations in young and aged mice were analysed by flow cytometry and IHC. Flow cytometric analysis demonstrated an increase in the representation and number of SCSM, medullary sinus macrophages and medullary cord macrophages in LN from aged mice (Fig. 2a,b). These results were confirmed by IHC. Aged mice displayed an increased depth of SCSM from the capsule (Fig. 2c), accompanied by an increase in macrophages in the medullary sinus and cord regions (Fig. 2c). Although blebbing of CD169 should be taken into account when considering the increase of SCSM^16^ both flow cytometry and IHC suggested a significant increase in the presence of these cells. These data show that the abundance of SCSM, medullary sinus macrophages and medullary cord macrophages is increased in aged LN.

### Aged LN have decreased numbers of stromal cells and decreased FDC networks

**Figure 3:**
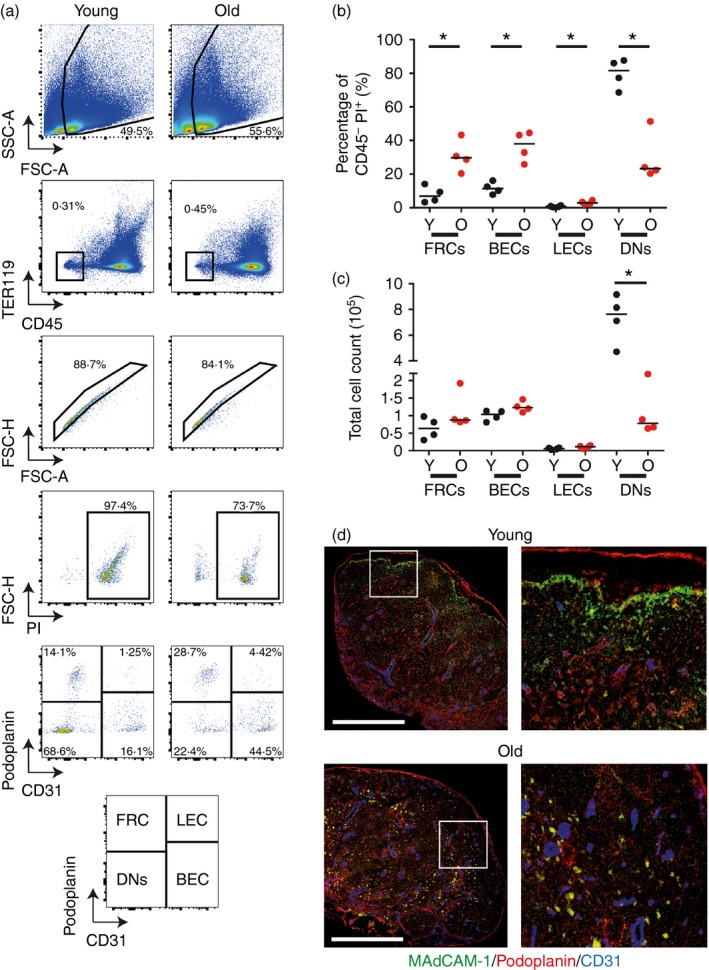
Aged lymph nodes (LN) have altered stromal cell representation and localization. Stromal cell representation and localization in the spleens of young and aged mice were analysed via flow cytometry and immunostaining. (a) Flow cytometry analysis of stromal cell populations in young and aged mice. Stromal cells were gated on forward and side scatter, intracellular propidium iodide staining and were CD45^−^, TER119^−^. The markers CD31 and podoplanin were used to identify fibroblastic reticular cells (FRC, podoplanin^+^,CD31^−^), lymphatic endothelial cells (LEC, podoplanin^+^, CD31^+^), blood endothelial cells (BEC, Podoplanin^–^, CD31^+^) and double negative (DN; Podoplanin^–^, CD31^−^). Results are indicative of three experimental repeats with three or four mice per group in each repeat. (b) Percentage of FRC, BEC, LEC and DN in young and aged LN. (c) Total cell counts of FRC, BEC, LEC and DN in young and aged LN. (d) Representative immunostaining of inguinal LN for MAdCAM‐1 (green), Podoplanin (red) and CD31 (blue). *P < 0·05. Scale bars represent 500 μm.

**Figure 4:**
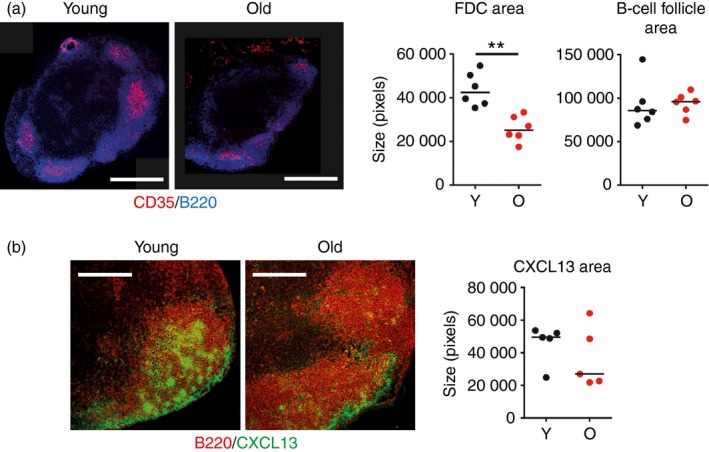
Decreased size and CXCL13 expression on aged follicular dendritic cells (FDC). (a) Histological sections of inguinal lymph nodes (LN) with immunostaining for B cells (B220 – blue) and FDC (CD35 – red) and measurement of the area of CD35^+^ FDC and B220^+^ B‐cell follicles in young and aged mice. Scale bars represent 500 μm. **P < 0·01. (b) CXCL13 staining (green) in the B‐cell follicles (red) of inguinal LN from young and aged mice and measurement of CXCL13 area in young and aged mice. Results are from five or six mice per group. Scale bars represent 200 μm.

Stromal cells are important for structural organization and immune responses in LN.^17^ Flow cytometry and IHC were used to characterize the effects of ageing on LN stromal cell populations. Stromal cells were isolated from pooled LN of young and aged mice through digestion of the LN with collagenase and analysed through flow cytometry. Stromal cells were identified by gating as detailed in Fig. 3(a). Forward and side scatter were used to remove detritus and doublets, a particular problem in the analysis of aged organs. CD45 and TER119 were then used to remove lymphocytes and red blood cells, respectively. Intracellular staining with PI was used to confirm that the populations analysed were true cells and not debris. Finally, the markers CD31 and podoplanin were used to identify four stromal cell populations as shown in the lower panels of Fig. 3(a). This analysis revealed a decrease in double‐negative (podoplanin^‐^CD31^–^) stromal cells in aged LN (Fig. 3a–c). IHC analysis of LN from young and aged mice also demonstrated altered localization of blood endothelial cells (BEC, CD31^+^) and FRC (podoplanin^+^) in aged LN (Fig. 3d). In aged mice the BEC and FRC were more widely distributed throughout the LN, contrary to their centralization around the efferent lymph draining side of the LN normally seen in young mice. Overall, BEC were abundant throughout the LN of aged mice and FRC localized around these. A decrease in the MAdCAM‐1^+^ marginal reticular cells was observed in approximately 50% of the aged mice analysed (Fig. 3d). Consistent with previous reports the FDC network size in aged mice was reduced (Fig. 4a)^8^, ^18^ but this did not affect the B‐cell follicle size (Fig. 4a). There was also less expression of the chemokine CXCL13 localized to the follicular region of the aged LN (Fig. 4b), in contrast to previously published data showing an increase in the LN with age.^10^ The loss of CXCL13^+^ marginal reticular cells correlated with their loss of MAdCAM‐1 and was also observed in approximately 50% of the aged LN.

### Aged SCSM and B cells display normal uptake and trafficking of antigen

We next determined whether the delivery of IC to FDC was adversely affected in the LN of aged mice. To do so, we adapted a previously characterized method,^1^ which enables the tracking of IC around the LN, from their arrival in the subcapsular sinus to their uptake by FDC. With this method, PE‐labelled immune complexes (PE‐IC) are initially acquired by SCSM, passed onto follicular B cells and subsequently deposited onto FDC. This technique therefore provides the opportunity to readily test the functioning of several LN populations in their ability to acquire and transport IC.

**Figure 5:**
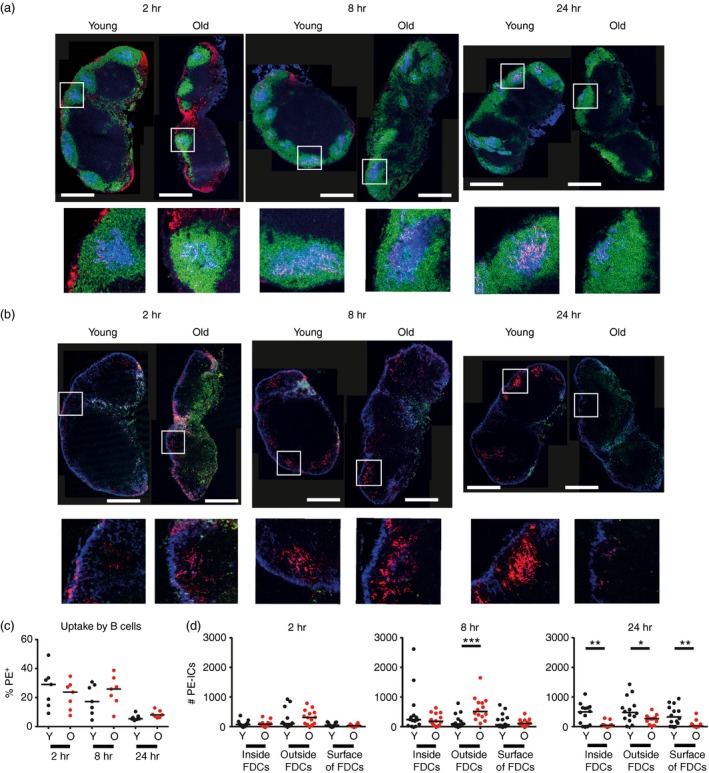
Follicular dendritic cells (FDC) fail to retain immune complexes in aged lymph nodes (LN). Young and aged mice were given polyclonal anti‐phycoerythrin intraperitoneally 16 hr before phycoerythrin was given subcutaneously into each side of the flank. Mice were killed 2, 8 and 24 hr after phycoerytherin injection and one inguinal LN was collected for immunostaining analysis while the other was used for flow cytometric analysis. This allowed tracking of phycoerytherin immune complexes (PE‐IC) from their uptake by subcapsular sinus macrophages (SCSM), their transference onto B cells, and through to deposition onto FDC. (a) Immunofluorescent staining of LN from young and aged mice 2, 8 and 24 hr post‐injection displays PE‐IC (red), B cells (B220^+^, green) and FDC (CD35^+^, blue). (b) Immunofluorescent staining of LN from young and aged mice 2, 8 and 24 hr post‐injection displays PE‐IC (red), SCSM (CD169^+^, blue) and other macrophages (F4/80^+^, green). (c) Flow cytometric analysis of inguinal LN was used to determine the percentage of B cells that have taken up PE‐IC (B220^+^,PE ^+^). (d) The number of PE‐IC inside, on the surface and more than 1 μm away from the surface (outside) of FDC in young and aged LN at the time‐points studied, determined via imaris analysis of *z*‐stack immunostaining. Results are pooled from two separate experiments, totalling seven mice per group. ***P < 0 .001; **P < 0·01; *P < 0·05. Scale bars represent 500 μm.

No impairment in the uptake of PE‐IC in aged mice compared with young mice was observed at 2 hr post‐injection (Fig. 5a,b). In mice from each age group, PE‐IC co‐localized with the SCSM around the edge of the LN, in the process of being transported to the FDC. However, the aged LN had larger deposition of PE‐IC within the inter‐follicular regions due to the previously detailed accumulation of macrophages (Fig. 2). Hence, aged mice displayed no initial impairment in the ability of SCSM to take up PE‐IC.

At 8 hr post‐injection no significant differences were observed in the localization of the PE‐IC between young and aged mice (Fig. 5a,b). Both age groups displayed deposition of PE‐IC on the edges of the FDC region. However, at 24 hr post‐injection, the young LN had a large accumulation of PE‐IC in the FDC region, whereas there were very few PE‐IC within the FDC region in any of the aged LN (Fig. 5a,b). Flow cytometric analysis of B‐cell populations demonstrated no significant difference at all three time‐points in the ability of young or aged B cells to acquire the PE‐IC (Fig. 5c), suggesting no alteration in the ability of aged B cells to acquire IC in aged LN.

### Decreased FDC size impairs IC uptake in aged LN

imaris software was next used to compare the relative abundance of PE‐IC inside the FDC, on the surface of the FDC and > 1 μm from the FDC surface (i.e. ‘outside’ the FDC; Fig. 5d) as adapted from a published protocol.^15^ Representative images of the *z*‐stacks obtained post‐analysis are provided in the Supplementary material (Fig. S1a). Although no significant changes were observed at 2 hr post‐injection in the abundance and distribution of the PE‐IC between age groups (Fig. 5d), at 8 hr post‐injection significantly more PE‐IC were present outside the aged FDC than those from young mice. Furthermore, by 24 hr post‐injection, aged FDC displayed significantly fewer PE‐IC inside, outside or on their surfaces (Fig. 5d). These data correlated with the IHC analysis, which showed limited retention of PE‐IC in the aged LN at 24 hr post‐injection (Fig. 5a,b). Analysis of FDC volume using imaris at each time‐point suggested the total volume of the aged FDC was reduced when compared with young FDC (see Supplementary material, Fig. S1b). Further analysis suggested that the ratio of the internalized PE‐IC to FDC volume was similar in each age group. (see Supplementary material, Fig. S1c). These data suggest that the impaired PE‐IC uptake in the LN of aged mice is a consequence of the decreased size of their FDC. Together these data demonstrate that aged LN have no deficiency in the initial uptake and transport of IC by SCSM and B cells, but the retention of IC is impaired due to the decreased size of their FDC.

## Discussion

In the current study we characterized the ageing‐associated changes to lymphocytes and macrophages within murine LN. These changes were also accompanied by significant structural reorganization of LN stromal cells, including FDC. SCSM are strategically positioned underneath the LN capsule to collect pathogenic material and antigens from the lymphatic fluid and aid its delivery to B cells and FDC. We observed no change in the ability of SCSM to perform this task in aged LN, nor were B cells impaired in their uptake of IC. However, the retention of IC by FDC was significantly decreased in aged LN 24 hr post‐injection. FDC are important in the maintenance of immune responses and GC and their decreased ability to retain IC may contribute to the failure of aged LN to produce GC equivalent to the same magnitude as those in young animals.

Stromal cells form the scaffold of the LN and are important in the localization, survival and immune responses of the leucocytes within.^17^, ^19^ Circulating lymphocytes enter the LN from the blood through high endothelial venules, composed of BEC. They can also enter the LN from the lymph through lymphatic endothelial cells (LEC). Once in the lymph node they localize to their associated T‐cell and B‐cell zones through movement along FRC and FDC, respectively,^4^ where they reside temporarily before relocating to other immune sites.^20^ Lymphocytes primarily leave the LN through the cortical sinus to enter the lymph^21^, ^22^, ^23^, ^24^ but can also exit the LN through LEC to enter the lymph.^25^ Although the total number of BEC, FRC and LEC in aged LN was unchanged, a decrease in the total number of double‐negative (DN) cells was observed. However, their distribution within the aged LN was grossly disorganized in aged mice, in a similar manner to that seen in the spleen.^26^ Stromal cells are important for the homeostasis and migration of T cells.^4^, ^27^ The structural disorganization seen with age may account for the decrease in T cells seen here, the impaired movement of naive T cells and B cells into the LN previously published,^9^, ^10^, ^11^ along with the decreased recruitment of leucocytes after infection.^12^ Furthermore, the population of DN (i.e. podoplanin^–^ CD31^−^) stromal cells was decreased in aged LN. This is not a well‐studied population and as such it is difficult to determine what population exactly is lost with age, and the implications this may have on host immunity. Furthermore, the method of LN digestion may impact on the representation of stromal cell populations. Different LN digestion protocols obtain wide‐ranging (10–60%) yields in the relative representation of this DN population.^10^, ^28^, ^29^ Regardless, independent transcriptional profiling of these DN cells has indicated that they express cytokines and chemokines that are important for survival, migration and localization of numerous leucocyte populations.^30^ Hence the loss of this population may have implications in the ability of aged LN to effectively recruit leucocytes and respond to pathogens.

Although the loss of FDC in aged LN has been demonstrated previously,^7^, ^8^, ^31^ the effect this has on B‐cell movement and responses appears to be minimal.^12^ Here we have demonstrated that B cells in aged LN have no deficiency in their ability to initially acquire IC and transport them to FDC. Aged B cells also appear to have no impairment in their ability to traffic to LN nor in their antibody responses, despite reductions in the expression of the B‐cell chemoattractant CXCL13.^12^ When acquired by FDC the IC undergo a cyclic process. The IC are first internalized and then periodically presented on the FDC surface before being re‐internalized.^3^ This process occurs over the period of a few hours and is considered to protect the antigens from damage while rendering them available for presentation in their native state to B cells. As demonstrated here, FDC within ageing LN are able to acquire and retain IC up to 8 hr post‐challenge to a similar extent to that observed in young LN. However, at 24 hr post‐injection very little IC was evident in the age LN, which was compounded by the decreased size of the FDC. Although the fate of these IC is unknown, it is plausible that they are degraded. What can be concluded is that without IC available for presentation to leucocytes, the immune response in the aged LN would be stunted. FDC are important in the retention of cells within GC.^32^ *In vitro* experiments have demonstrated that aged FDC had reduced trapping and presentation of antigen to B cells, which could be restored with the administration of complement.^13^ The lack of IC retention by aged FDC, due to their decreased size, may help to explain why GC responses are impaired in aged mice^7^, ^8^, ^33^ and provide a target for future therapeutic intervention to improve immunity in the elderly. Hence, our data provide an important advance in our understanding of why immune responses fail in aged LN and why the elderly have decreased responses to vaccination and increased susceptibility to viral and bacterial infections.

## Author contributions

V.M.T. performed the experiments. V.M.T. and N.A.M. designed the experiments, interpreted the data and wrote the manuscript.

## Disclosures

The authors report no conflicts of interest.

## Supporting information


**Figure S1.** Imaris analysis of phycoerythrin–immune complex localization on follicular dendritic cells.
