# Supplementary figures and images for: Structural and functional changes to lymph nodes in ageing mice
Source: Immunology. 2017 Mar 16;151(2):239–47. doi: 10.1111/imm.12727 (PMC5418465; doi:10.1111/imm.12727)

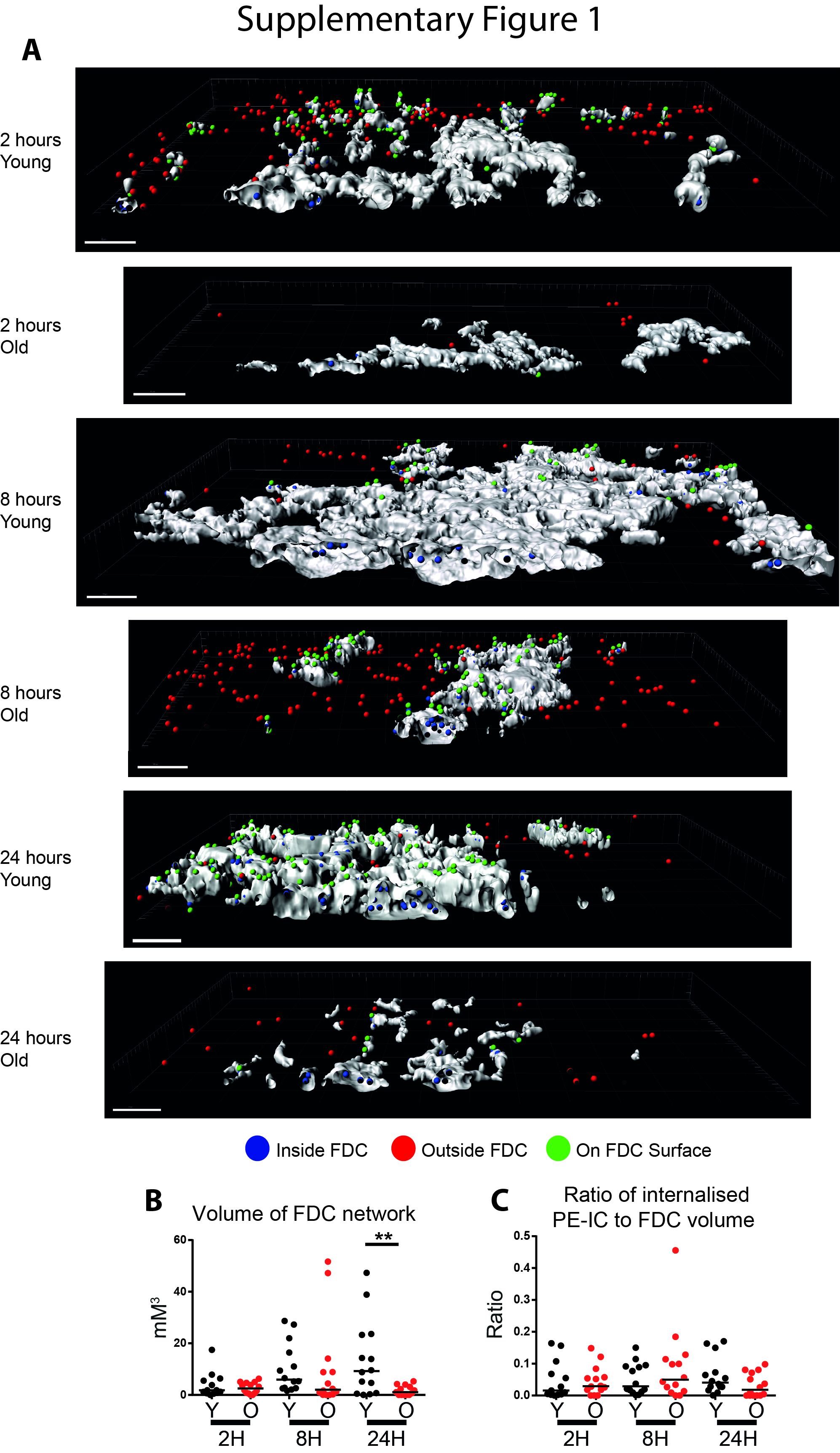

Supplement: Supplementary file 1 — Figure S1. Imaris analysis of phycoerythrin–immune complex localization on follicular dendritic cells. [file IMM-151-239-s001.tif]
